# Effects of LED Spectra on Biochemical and Histological Profiles of *Eurycoma longifolia* Hairy Root Cultures

**DOI:** 10.21315/tlsr2026.37.3.13

**Published:** 2026-10-15

**Authors:** Mahmoud Ali Khalaf Abushattal, Sani Sale, Sreeramanan Subramaniam, Nor Hasnida Hassan, Mohamad Fadhli Mad’ Atari

**Affiliations:** 1 School of Biological Sciences, Universiti Sains Malaysia, 11800 USM, Pulau Pinang, Malaysia; 2 Department of Genetic Engineering and Biotechnology, Faculty of Allied Medical Sciences, Middle East University, Amman, Jordan; 3 Department of Botany, Gombe State University, Gombe P.M.B 127, Nigeria; 4 Centre for Chemical Biology (CCB), Universiti Sains Malaysia, Bayan Lepas, 11900, Pulau Pinang, Malaysia; 5 Department of Biology, Faculty of Science and Technology, Universitas Airlangga, Surabaya 60115, Indonesia; 6 Forest Biotechnology Division, Forest Research Institute Malaysia (FRIM), 52109 Kepong, Selangor, Malaysia

**Keywords:** *Eurycoma longifolia*, Hairy Root Culture, Histological, Antioxidant, Protein Content, Eurycoma longifolia, Kultur Akar Berambut, Histologi, Antioksidan, Kandungan Protein

## Abstract

The roots of *Eurycoma longifolia* Jack, commonly used in traditional Malaysian medicine, are known for their aphrodisiac properties and anti-cancer potential. Hairy root culture (HRC) represents a sustainable and promising platform for the enhanced production of secondary metabolites. This study explored the impact of different light-emitting diode (LED) treatments on *Elongifolia* hairy root cultures (ELHRCs) over 12 weeks. Five LED spectra were used: red (R), blue (B), mint green (MG), blue plus red in a 1:1 ratio (BR) and white (W, as control). The study aimed to evaluate the effects of these light treatments on quantified histological characteristics, total carbohydrate and protein content, and antioxidant activity of ELHRCs at 8 weeks, 10 weeks and 12 weeks of culture. Quantitative five-directional radial histological analysis revealed notable tissue repartitioning; specifically, the MG treatment exhibited the highest cortical expansion (~400 μm) alongside a reduction in vascular radius relative to the W control. These structural modifications corresponded to distinct biochemical shifts. The results showed that the W control and BR light treatment significantly maintained the highest protein content across all culture durations, with maximum concentrations of 0.707 ± 0.07 mg/mL and 0.706 ± 0.01 mg/mL, respectively. Meanwhile, compared to the W control, R and B light treatment promoted higher carbohydrate accumulation, particularly after 8 weeks and 12 weeks, with values reaching 116.79 ± 4.34 mg/mL and 103.68 ± 15.15 mg/mL. Antioxidant activity, measured through DPPH radical scavenging, was nominally highest under R light after 8 weeks and 10 weeks (92.13% and 90.28%, respectively), though these values were not significantly different from the W control. These findings highlight the potential of LED treatments to optimise metabolite production in hairy root cultures, offering insights into improving bioactive compound yield for pharmaceutical and nutraceutical applications.

## Abstrak

Akar Eurycoma longifolia Jack, yang lazim digunakan dalam perubatan tradisional Malaysia, terkenal dengan sifat afrodisiak dan potensi antikansarnya. Kultur akar berambut (Hairy Root Culture, HRC) merupakan platform yang lestari dan menjanjikan untuk penghasilan metabolit sekunder yang dipertingkatkan. Kajian ini meneroka kesan rawatan cahaya daripada diod pemancar cahaya (Light Emitting Diodes, LED) yang berbeza terhadap kultur akar berambut E. longifolia (ELHRC) selama 12 minggu. Lima spektrum LED telah digunakan: merah (R), biru (B), hijau pudina (MG), gabungan biru dan merah pada nisbah 1:1 (BR) dan putih (W, sebagai kawalan). Kajian ini bertujuan menilai kesan rawatan cahaya terhadap ciri histologi kuantitatif, kandungan protein dan karbohidrat keseluruhan, serta aktiviti antioksidan ELHRC pada minggu ke-8, ke-10 dan ke-12 kultur. Analisis radial lima arah secara kuantitatif menunjukkan pembahagian semula tisu yang ketara; secara khusus, rawatan MG mempamerkan pengembangan korteks tertinggi (~400 μm) seiring dengan pengurangan jejari vaskular berbanding dengan kawalan W. Perubahan struktur ini selari dengan anjakan biokimia yang dikesan. Keputusan menunjukkan bahawa cahaya kawalan W dan rawatan BR meningkatkan kandungan protein dengan ketara sepanjang tempoh kultur, dengan kepekatan maksimum masing-masing sebanyak 0.707 ± 0.07 mg/mL dan 0.706 ± 0.01 mg/mL. Sementara itu, berbanding kawalan W, rawatan cahaya R dan B merangsang pengumpulan karbohidrat yang lebih tinggi, terutamanya pada minggu ke-8 dan ke-12, dengan nilai mencapai 116.79 ± 4.34 mg/mL dan 103.68 ± 15.15 mg/mL. Aktiviti antioksidan, yang diukur melalui keupayaan menyingkir radikal DPPH, adalah tertinggi secara nominal bawah cahaya R selepas minggu ke-8 dan ke-10 (masing-masing 92.13% dan 90.28%), namun keputusan ini tidak signifikan berbanding dengan kawalan W. Penemuan ini menyerlahkan potensi rawatan LED untuk mengoptimumkan penghasilan metabolit dalam kultur akar berambut, serta memberikan pandangan baharu dalam usaha meningkatkan hasil sebatian bioaktif untuk aplikasi farmaseutikal dan nutraseutikal.

**HIGHLIGHTS**

- LED spectral quality significantly modulates the histological repartitioning and biochemical profiles of *Eurycoma longifolia* hairy root cultures.
- Red and blue LED spectra promote significantly higher carbohydrate accumulation (up to 1.93-fold) compared to the white light control.
- Quantitative anatomical analysis reveals that mint green light induces maximal cortical expansion, while red light maintains the highest antioxidant stability.

## INTRODUCTION

*Eurycoma longifolia* is a traditional Malaysian medicinal plant and known as “Tongkat Ali” in Malaysia. It’s a medium-sized tree that typically reaches about 15 m to 18 m in height and belongs to the Simaroubaceae family (Nordin 2014). The root extract of *E*. *longifolia* is highly valued for its aphrodisiac properties (Bhat & Karim 2010) and its efficacy against cardiovascular diseases (Al-Joufi *et al*. 2017). Quassinoids such as eurycomanone and alkaloids such as 9-methoxycanthin-6-one are the two main groups of bioactive components in *E. longifolia* (Rehman *et al*. 2016). These compounds exhibit anti-inflammatory (Hien *et al*. 2019), anti-cancer (Yunos *et al*. 2022; Sani *et al*. 2024), anti-parasite (Abushattal *et al*. 2025b), antioxidant (Varghese *et al*. 2013), anti-tumour (Bhat & Karim 2010) and anti-malarial activities (Kuo *et al*. 2004). Some of these medicinal properties have been proven using clinical trials, thus giving *E*. *longifolia* additional popularity (Thu *et al*. 2018; Chaingam *et al*. 2021; Al-Bayati *et al*. 2022; Sale *et al*. 2024). However, these phytochemicals can play an important role in therapeutic medication strategies for a wild range of diseases, especially in cancer treatment (Saadh *et al*. 2026).

To ensure a sustainable supply of *E. longifolia*, hairy root cultures (HRCs) serve as an alternative production system due to their high growth rate and ability to biosynthesise secondary metabolites. Hairy roots are induced by *Rhizobium rhizogenes* transformation and have been widely utilised in plant biotechnology for metabolite production (Gutierrez-Valdes *et al*. 2020). Enhancing the yield of valuable bioactive compounds in hairy root cultures requires optimisation of growth conditions, including media composition and elicitation techniques (Georgiev *et al*. 2012). Previous studies have shown that methyl jasmonate and salicylic acid can enhance 9-methoxycanthin-6-one production (Nazirah *et al*. 2018), while yeast elicitor has been used to increase eurycomanone content (Nhan & Loc 2018). Furthermore, specialised bioreactor systems have been explored to scale up biomass production (Natanael *et al*. 2014; Fan *et al*. 2021). However, limited studies have explored alternative approaches, such as light treatments, for improving the biosynthesis of secondary metabolites in *E. longifolia* hairy root cultures (Sale *et al*. 2024).

Light quality plays a crucial role in plant morphogenesis, growth and metabolic activity (Araújo *et al*. 2021; Nery *et al*. 2021). The use of light-emitting diodes (LEDs) offers precise spectral control, enabling targeted regulation of *in vitro* plant development (Jung *et al*. 2021). LEDs are advantageous due to their ability to provide specific wavelengths that are efficiently absorbed by plant photoreceptors, influencing root growth and secondary metabolite production (Batista *et al*. 2018; Chaves *et al*. 2020; Paradiso & Proietti 2022). For instance, combined red and blue LEDs have been shown to improve root biomass and carbohydrate biosynthesis in *Doritaenopsis* (Shin *et al*. 2008), while blue light increased protein content in *Achillea millefolium* (Alvarenga *et al*. 2015). In addition, blue LEDs irradiation was found to promote astragaloside production in *Astragalus membranaceus* hairy root cultures with biosynthetic gene expression (Gai *et al*. 2023), which indicates that light plays an essential role in secondary metabolism.

Biochemical and histological analyses are essential for understanding the impact of LED treatments on *E. longifolia* hairy root cultures. Environmental stress factors, such as light exposure, can induce physiological changes affecting membrane fluidity, protein conformation, and nucleic acid integrity (Chinnusamy *et al*. 2007). The 1,1-diphenyl-2-picrylhydrazyl (DPPH) assay is widely used to assess antioxidant activity, providing insights into plant responses to oxidative stress (Alam *et al*. 2013; de Torre *et al*. 2019). Histological analysis plays a critical role in evaluating cellular structures, tissue organisation and secondary metabolite accumulation in plant tissues (Croteau *et al*. 2000; Govindaraju & Indra Arulselvi 2018). As described by Croteau *et al*. (2000) the integration of anatomical data is vital for mapping the metabolic sites of specialised compounds. It allows for the visualisation of anatomical modifications induced by different growth conditions and can serve as a tool for pre-analysis of metabolite localisation and post-analysis of structural changes in response to LED treatments (Dobrowolska *et al*. 2017; Ferreira *et al*. 2017).

Despite the promising effects of LED spectra on various plant systems, no studies have investigated their impact on *E. longifolia* hairy root cultures (ELHRCs). In particular, there is limited knowledge about how LED treatments influence carbohydrate and protein content, antioxidant activity, and histological characteristics in ELHRCs. Therefore, this study aims to explore the effects of different LED spectra on the biochemical profiles and histological properties of ELHRCs compared to a white light control.

## MATERIALS AND METHODS

### Plant Material and Growth Conditions

The ELHRCs used in this study were obtained from the Forest Research Institute Malaysia and maintained at the School of Biological Sciences, Universiti Sains Malaysia. The cultures were grown in Murashige and Skoog (MS) basal medium, with the pH adjusted to 5.8 and sterilised by autoclaving at 121°C for 15 min. The cultures were incubated at 22 ± 2°C on an orbital shaker set at 110 rpm under a controlled light regime with a 16-hour light and 8-hour dark photoperiod.

To evaluate the effects of different LED spectra on the histological characteristics, biochemical properties and antioxidant activity of ELHRCs, five light treatments were applied: white (400 nm–700 nm) [W], blue (440 nm) [B], red (660 nm) [R], blue plus red in a 1:1 ratio (440 nm + 660 nm) [BR] and mint green (530 nm) [MG]. The photosynthetic photon flux densities (PPFD) of the LEDs were measured as follows: W (44.38 μmol m^−2^ s^−1^), R (14.24 μmol m^−2^ s^−1^), B (6.40 μmol m^−2^ s^−1^), BR (17.63 μmol m^−2^ s^−1^) and MG (7.90 μmol m^−2^ s^−1^). White light served as the control treatment.

Hairy root samples were collected at three intervals (8 weeks, 10 weeks and 12 weeks) from each light treatment and analysed for histological characteristics, total carbohydrate and protein content, and antioxidant activity. The experimental setup followed previously reported methodologies (Abushattal *et al*. 2025a).

### Histological Analysis

At 8 weeks, 10 weeks and 12 weeks of culture, roots from three independent flasks per treatment were pooled to form a representative composite sample. From this pooled sample, multiple root tips (1.0 cm in length) were randomly selected for histological analysis to ensure representative observations of the treatment effects compared to the white light control. The samples were carefully removed and immersed in a FAA solution (formalin, 95 % acetic acid and 95 % alcohol (ethanol) at a ratio of 1:1:1) for at least 24 h. This fixation process was carried out at a temperature of 4°C. The samples were immersed in a solution of tertbutyl alcohol (TBA) with concentrations of 50%, 70%, 85% and 100% in different gradations, followed by successive treatments with xylene and Paraplast Plus® wax (Sigma-Aldrich) in a 1:1 ratio at 60ºC (Memmert, Germany), followed by a threefold exchange of the solution with fresh paraffin wax before blocking with an embedding device (Leica EG 1160, Germany). The samples were then sectioned with a microtome (Leica RM2135) at a thickness of 9 μm. Sections were stained overnight with Safranin for an extended period of time, followed by a shorter 15-minute staining with Fast Green. The slides were then examined under a compound light microscope (SIS Image Analyser, Olympus). Quantitative histological analysis was performed using ImageJ software (ImageJ 1.54 g). For each treatment, five radial measurements were taken from the centre of the stele to the endodermis and from the endodermis to the epidermis in five different directions. Data are presented as the Mean ± Standard Error of these five spatial replicates to account for the anatomical variance within the pooled root samples.

### Biochemical Analysis

#### Protein extraction

Total soluble proteins were isolated from 100 mg fresh weight of ELHRCs from 8 weeks, 10 weeks and 12 weeks of all treatments (using samples of three independent flasks) in which the roots were crushed with a pre-cooled mortar and pestle and homogenising with 0.6 mL of an enzyme extraction buffer solution. The buffer solution for enzyme extraction was prepared from 100 mM potassium phosphate buffer (pH 7.8), 2 mM ethylenediaminetetraacetic acid (EDTA) (Sigma-Aldrich, USA) and 2.0% (w/v) polyvinylpyrrolidone (PVP) (Fluka Chemie, Switzerland) according to Monnet *et al*. (2006).

The extracts of hairy roots were purified by centrifugation at 15,000 ×g for 20 min (Gyrozen, model 1524). The resulting supernatants were used for the determination of soluble proteins. The extract solution with a volume of 0.1 mL was added to 1.0mL Bradford reagent and vortexed. After 5 min, the absorbance was measured at 595 nm using a UV-visible microplate spectrophotometer (Thermo Scientific™ Multiskan™ GO). The soluble protein concentration was calculated using the standard curve generated with bovine serum albumin (BSA) at concentrations of 0 μg/mL, 200 μg/mL, 400 μg/mL, 600 μg/mL, 800 μg/mL and 1,000 μg/mL, which was used as a standard according to Le and Stuckey (2016) with modification.

#### Carbohydrate extraction

A total of 100 mg of the frozen ELHRCs from 8 weeks, 10 weeks and 12 weeks of culture (using samples of three independent flasks) in which the roots were crushed into small pieces using a mortar and pestle, placed in a 25 mL test tube containing 15mL of 80 % (v/v) ethanol and extracted by incubation in a 60°C water bath for 30 min. In the meantime, 2.0 mL of 2.0% anthrone reagent was added to a clean test tube and kept on ice. Once the hairy root was extracted, it was filtered using Whatman No. 1 filter paper. The filtrates were then added dropwise against the wall into the anthrone-containing test tubes to avoid blackening of the samples. The anthrone-filtrate mixture was then further incubated in a 95°C water bath for 15 min. The test tubes were removed from the water bath and placed in a cold bath to stop the reaction immediately. The absorbance of the samples was recorded at 630 nm and the carbohydrate content was estimated using the standard glucose curve (0 mg/mL, 200 mg/mL, 400 mg/mL, 600 mg/mL mg/mL, 800 mg/mL and 1,000 mg/mL according to Kruger (2009) with modification.

#### Determination of antioxidant activity

After 8 weeks, 10 weeks and 12 weeks of cultivation, a sample weight of 200 mg was determined by weighing the *in vitro* hairy roots of all treatments (using samples of three independent flasks). These samples were crushed in 1.2 mL of 80 % (v/v) acetone using a pre-cooled mortar and pestle. The samples were transferred to 25 mL culture vessels with black lids and stored overnight at 4°C in the dark. Antioxidant activity was measured by the ability of the plant extracts to neutralise free radicals. The free radical 2,2-diphenyl-1-picrylhydrazyl (DPPH) was used to investigate the free radical scavenging activity of ELHRCs according to the protocol of Shen *et al*. (2010).

DPPH at a concentration of 0.1 mM in acetone was freshly prepared one day before the experiment. At the beginning of the experiment, 1.0 mL of the solution was mixed with 1.0 mL of the prepared extracts of hairy roots in acetone after centrifuging the samples at 13,000 ×g for 5 min. The mixtures were allowed to stand for 30 min at room temperature in the dark to allow the extract to react with the DPPH radicals. The absorbance of the mixture was measured with a UV/vis microplate at 517 nm using ascorbic acid as a reference. The ability of the sample extract to scavenge DPPH radicals was determined using the equation given by Shen *et al*. (2010):


$$\text{Percentage }(\%)\;\text{inhibition}=[({\text{A}}_{517})-({\text{A}}_{\text{Sample}})]/[({\text{A}}_{517})]\times 100$$

Where A517 = absorbance of DPPH radical + acetone at 517 nm and A sample = absorbance of DPPH radical + sample extract/ reference.

### Statistical Analysis

All experiments for biochemical and antioxidant assays were performed in three replicates. The data were analysed using a (one-way) analysis of variance (ANOVA) followed by Duncan’s post hoc test at *p* ≤ 0.05 using the Statistical Package for the Social Sciences (SPSS) program (version 28). For histological analysis, due to the representative nature of the micrographs, a descriptive quantitative approach was used. Data are expressed as the mean ± standard error (SE) derived from five radial spatial replicates per treatment to account for anatomical variance. The white light (W) group was utilized as the control treatment for all comparisons. The results were expressed as mean values and their standard errors (SE) using MS Excel software.

## RESULTS AND DISCUSSION

### Comparative Histological Characterisation of LED-treated ELHRC*s*

As detailed in Table 1, the quantitative 5-directional analysis revealed notable tissue repartitioning over the 12-week period. While the White (W) control exhibited a stable cortical width (~371 μm by week 12), the Mint Green (MG) treatment exhibited a notably thicker cortex (~400 μm) at week 12, as measured across multiple radial axes. However, this expansion in MG was accompanied by a reduction in vascular radius (~64 μm) compared to the W control (~100 μm), suggesting a metabolic prioritisation of cortical tissue expansion over vascular core development under green light spectra. These results confirm the differences in thickness and texture qualitatively observed in Fig. 1. The histological analysis revealed distinct structural differences between the W control group and LED-treated ELHRCs, depending on treatment duration (Fig. 1 A–E). While all roots initially exhibited a defined epidermis and central vascular column, monochromatic light altered the tissue proportions relative to the W control. Compared to the W control, notable structural modifications were observed in response to LED treatments, particularly after 8 weeks, 10 weeks and 12 weeks. Specifically, by week 10, MG-treated roots showed a 15.3% increase in cortical width compared to the W control, while Red (R) light induced a more compact cortical arrangement with a narrower width (~232 μm).

After 8 weeks, hairy roots treated with R, B and the W control exhibited compact epidermal cells that elongated in the radial direction. In contrast, plasmolysis, characterised by the presence of dark-stained content within vacuoles and cytoplasm, was qualitatively evident in MG-treated hairy roots coincided with a measured reduction in vascular radius compared to the W control (Table 1). After 10 weeks, modest histological differences emerged between the control and LED-treated hairy roots, with plasmolysis detected in the W control and MG-treated hairy roots. After 12 weeks, the most pronounced structural changes were observed, where epidermal cells became densely packed and elongated in the tangential direction, forming concentric layers. However, the results showed no clear variation in vacuolisation and pigmentation between treatments. These anatomical variations are consistent with our previous reports (Sale *et al*. 2024; (Abushattal *et al*. 2025a; 2025b).), which demonstrated that specific LED spectra influence ELHRCs growth while maintaining high genetic stability (90.3%–100%), confirming these changes are physiological adaptations rather than spectral-induced mutations.

In a similar study, hairy roots were found to have closely packed and elongated epidermal cells and ellipsoidal cortical cells (Dmitrović *et al*. 2015). Furthermore, histological examination of *Eucalyptus* hairy roots revealed a star-shaped xylem structure with increased lignified tissues, suggesting secondary growth with lignin deposition (Plasencia *et al*. 2016). This aligns with our observations of the robust central stele in ELHRCs, which remained structurally connected to the cortical tissues across LED treatments. Similarly, less densely packed cortical cells with intercellular cavities were observed in other hairy roots (Gavrilovic *et al*. 2016; Triozzi *et al*. 2021).

These morphological alterations indicate structural adaptations to varying light spectra, potentially influencing nutrient absorption and secondary metabolite accumulation. It was demonstrated previously that MG and R light significantly enhance the synthesis of quassinoids (eurycomanone) and anti-parasitic alkaloids (9-Methoxycanthin-6-one) in these specific ELHRC lines (Sale *et al*. 2024; Abushattal *et al*. 2025b). The observed structural repartitioning likely reflects the metabolic “remodelling” required to facilitate the increased yields of these bioactive compounds. The presence of plasmolysis in specific treatments suggests possible osmotic stress or metabolic responses, aligning with previous reports on LED-induced physiological changes in plant tissue cultures (Araújo *et al*. 2021; Nery *et al*. 2021).

After 12 weeks of culture, both the W control and LED-treated hairy roots exhibited densely packed epidermal cells with a high degree of vacuolization in the maturation zone (Fig. 1 A–E). The cortical cells displayed vacuolated structures with intercellular cavities and varied in shape and size. Additionally, the central stele of the vascular tissue maintained a strong connection with the surrounding cortical tissue. However, irregularities in phloem and xylem were frequently observed, including the expansion of intercellular gaps, and a loose connection between the central stele and cortical cells (Figs. 1A, 1C and 1E). This tangential reorientation of the epidermis suggests a protective mechanism for the internal tissues under spectral stress.

Similar anatomical variations in response to light exposure have been documented in other hairy root systems. Mukherjee *et al*. (2014) reported that continuous illumination induced photooxidative stress in *Daucus carota* hairy roots, leading to increased vacuolisation and structural shrinking. This explains our findings in the W and MG treatments, where plasmolysis suggests that these specific spectra act as stressors that trigger cellular modifications. This observation aligns with our findings, as structural modifications were evident in ELHRCs exposed to different LED treatments. Moreover, Mukherjee *et al*. (2016; 2019) demonstrated that green hairy roots exhibited anatomical modulations, including an expanded vascular area to support light-induced growth; similarly, our ELHRCs maintained strong connectivity between the stele and cortex, suggesting an optimised vascular organisation to facilitate nutrient transport under LED stress.

Comparable variations in hairy root anatomy have also been reported in other plant species. Park and Facchini (2000) observed distinct differences in the organisation of epidermal cells and the development of transformed hairy roots in *Papaver somniferum* and *Eschscholzia californica*, noting that epidermal organisation is highly plastic in response to external stimuli. Similarly, Dmitrović *et al*. (2015) documented morphological differences in *Chenopodium murale* hairy roots, highlighting how environmental factors like light quality influence root density. These histological modifications confirm that LED spectra act as a morphogenic signal, specifically targeting cortical and vascular repartitioning to accommodate metabolic shifts, as evidenced by the anti-parasitic and anti-cancer activity reported in our previous extracts (Sale *et al*. 2024; Abushattal *et al*. 2025b).

### Effects of LED Treatment on Protein and Carbohydrate Content in Relation to Histological Changes in ELHRCs

The results in Table 2 indicate that different LED treatments significantly influenced protein and carbohydrate content in ELHRCs over time when compared to the white light (W) control. After 8 weeks, roots cultured under R, B and MG light exhibited significantly lower protein content compared to W control and BR light. Specifically, protein levels under R (0.271 mg/mL) and B (0.298 mg/mL) were approximately 60% lower than the W control (0.707 mg/mL). By week 12, the significant protein reductions remained most pronounced in R (0.234 mg/mL) and B (0.275 mg/mL) treatments, which were significantly lower than the W control (0.647 mg/mL) according to Duncan’s test. These variations suggest that monochromatic R and B wavelengths may inhibit protein metabolism in ELHRCs, possibly linked to the diversion of resources toward secondary metabolism. The decline in protein under monochromatic R and B light at week 8 and week 12 aligns with the high metabolic demand for alkaloid biosynthesis previously reported in these specific cultures (Abushattal *et al*. 2025b).

Histological examination revealed that roots under R, B and MG light at 8 weeks exhibited compact epidermal cells elongated in the radial direction. This structural adaptation may indicate stress-induced metabolic adjustments, potentially linked to lower protein accumulation as resources are diverted to structural reinforcement. Furthermore, plasmolysis was evident in BR- and MG-treated hairy roots at 8 weeks and in the W control and MG-treated hairy roots at 10 weeks. As noted in previous section (Comparative Histological Characterization of LED-treated ELHRCs), the structural repartitioning in the MG treatment (Table 1) provides anatomical evidence for the cellular response occurring under green light stress.

Carbohydrate content also showed dynamic responses to LED treatments relative to the W control. At 8 weeks, B and R lights led to significantly higher carbohydrate accumulation compared to the W control light, possibly reflecting increased photosynthetic efficiency or energy storage. However, after 10 weeks, the highest carbohydrate content was observed under R light, while B, BR and MG-treated hairy roots showed reduced carbohydrate levels. This decrease could be attributed to increased metabolic activity, supporting structural growth or secondary metabolite synthesis (Jung *et al*. 2021). After 12 weeks, carbohydrate accumulation was significantly higher under R, B, BR and MG lights compared to the W control, with R-treated hairy roots showing the greatest increase (1.93 times higher than the W control). This significant carbohydrate accumulation (116.79 mg/mL under red light) likely provides the carbon skeletons required for the enhanced biosynthesis of specialised metabolites observed in our previous studies (Sale *et al*. 2024; Abushattal *et al*. 2025b), as elevated primary carbon pools often precede secondary metabolite flux (Georgiev *et al*. 2012).

These biochemical findings align with histological observations, where the MG treatment exhibited the most significant cortical expansion (~400 μm, Table 1) despite moderate carbohydrate levels, suggesting high metabolic efficiency. Such structural modifications suggest that specific wavelengths enhance resource allocation, influencing both primary metabolism (protein and carbohydrate content) and tissue organisation. The increased carbohydrate content under R and B light treatments may have supported cellular expansion, vascular differentiation, and secondary metabolite biosynthesis, contributing to the overall structural and metabolic adaptation of ELHRCs under different LED treatments.

Previous studies support this trend. Shin *et al*. (2003) reported that sugar concentrations were highest in *Beta vulgaris* hairy roots grown under B and RB light, while R and far-red light led to the lowest accumulation. Similarly, Fan *et al*. (2022) found that *Dendrobium denneanum* plants exposed to B light for 60 days exhibited increased flavonoid content, whereas extending exposure to 120 days enhanced polysaccharide accumulation. Veremeichik *et al*. (2023) observed that BR light significantly increased anthocyanin content in *Eruca sativa*, while Szopa *et al*. (2018) found that combined B and R LEDs enhanced phenolic acid and flavonoid synthesis in several shoot cultures.

Light quality also influences carbohydrate and protein metabolism in various tissue cultures. Mengxi *et al*. (2011) demonstrated that R light promoted carbohydrate accumulation, while B light enhanced protein content in *Oncidium* protocorm-like bodies. Similarly, Li *et al*. (2010) showed that a combination of R and B light significantly affected physiological traits in *Gossypium hirsutum* tissue cultures, where R light maximised root activity, sucrose, starch and soluble sugar content. Amitrano *et al*. (2018) further found that RB light increased protein levels in *Vigna radiata*.

In line with these findings, the current study highlights the regulatory role of B and R light in modulating carbohydrate and protein content in ELHRCs, depending on treatment duration. The observed shifts in biochemical composition under different LED wavelengths suggest that light-driven metabolic changes play a crucial role in the synthesis of antioxidants and secondary metabolites. These results support the strong link between light quality, biochemical regulation and secondary metabolite production in ELHRCs. By linking these biochemical shifts to the structural repartitioning of the cortex and vascular tissues (Table 1) and the high genetic stability of the cultures (Abushattal *et al*. 2025a), these results confirm the potential of LED spectra as a powerful tool for eliciting high-value bioactive compounds in ELHRCs.

### Determination of the Effect of LED Treatment on Antioxidant Activity Analysis in ELHRCs

The results of DPPH radical scavenging activity in ELHRCs showed significant variations under different LED treatments across culture durations when compared to the white light (W) control (Fig. 2). After 8 and 12 weeks, antioxidant activity was significantly lower under B light (44.31% at week 12) compared to the W control (60.61%) and other LED treatments (Fig. 2). Conversely, while nominal antioxidant capacity was higher under R, BR, and MG light after 8 weeks and 12 weeks, these values remained statistically comparable to the W control baseline (Fig. 2). After 10 weeks, R and BR treatments induced nominally greater antioxidant activity compared to the W light control, although the differences were not statistically significant (*p* > 0.05) based on (Fig. 2). These results highlight the influence of specific LED wavelengths on antioxidant metabolism in ELHRCs, aligning with previous studies on light-mediated physiological responses.

The interplay between LED wavelengths, primary metabolism and histological characteristics provides insight into these observations. The earlier findings (Table 2) demonstrated that B and R light enhanced carbohydrate content at 8 weeks and 12 weeks, while W and BR light promoted protein accumulation at 8 weeks and 10 weeks. The increased carbohydrate content under R light may contribute to the antioxidant stability by providing precursors for secondary metabolite biosynthesis, particularly phenolic compounds and flavonoids. Indeed, our previous work confirmed that R light elicitation significantly enhances the yield of bioactive quassinoids (Sale *et al*. 2024), which likely contributes to the robust radical scavenging efficiency observed here. Similarly, the increased protein content under W and BR light suggests enhanced enzymatic activity, potentially supporting antioxidant defense mechanisms. The histological analysis further supports this relationship; specifically, the structural integrity and stable cortical width maintained under R light (Table 1) correlate with higher antioxidant stability, whereas the plasmolysis and cortical contraction observed under the W control and MG light (section Comparative Histological Characterisation of LED-treated ELHRCs) likely indicate photooxidative stress that compromises scavenging efficiency over time (Mukherjee *et al*. 2014).

Our findings are in agreement with Karimi *et al*. (2022), who reported that a combination of R and B light increased antioxidant enzyme activity and flavonoid content in *Hypericum perforatum* plants. Similarly, Jiao *et al*. (2015) demonstrated that UV-B elicitation significantly enhanced the antioxidant activity of *A. membranaceus* hairy root extracts. The wavelength-specific modulation of metabolic pathways has also been reported by Mirzahosseini *et al*. (2020), who observed distinct protein expression profiles in *Arabidopsis* plants under different light treatments. In this study, W, BR and R light maintained high antioxidant activity in ELHRCs, possibly due to the synergistic effect of B and R light, which is known to regulate various physiological functions and secondary metabolite biosynthesis in plants. These biochemical shifts are also reflected in the high genetic stability (90.3%–100%) and potent anti- -parasitic activity previously reported for these ELHRC extracts (Abushattal *et al*. 2025a; 2025b).

Collectively, these findings suggest that the antioxidant potential of ELHRCs is intricately linked to light-regulated biochemical and histological changes. The sustained antioxidant activity under W, R, BR and MG light correlates with enhanced protein and carbohydrate content, supporting the role of LED spectral quality in modulating plant defense responses and metabolic pathways.

## CONCLUSIONS

This study demonstrates that LED spectral quality significantly influences the biochemical and histological profiles of ELHRCs. Compared to the W control, the BR light treatment. consistently maintained protein content, whereas R and B lights induced the highest carbohydrate accumulation, particularly at 8 weeks and 12 weeks. Antioxidant activity exhibited wavelength-specific responses, with sustained high capacity under W, R, BR and MG light. Longitudinal histological modifications, specifically the cortical expansion under MG and structural stability under R light (Table 1), correlated with biochemical changes. Overall, this study underscores the potential of LED wavelengths to modulate secondary metabolite production in ELHRCs.

## Figures and Tables

**FIGURE 1:**
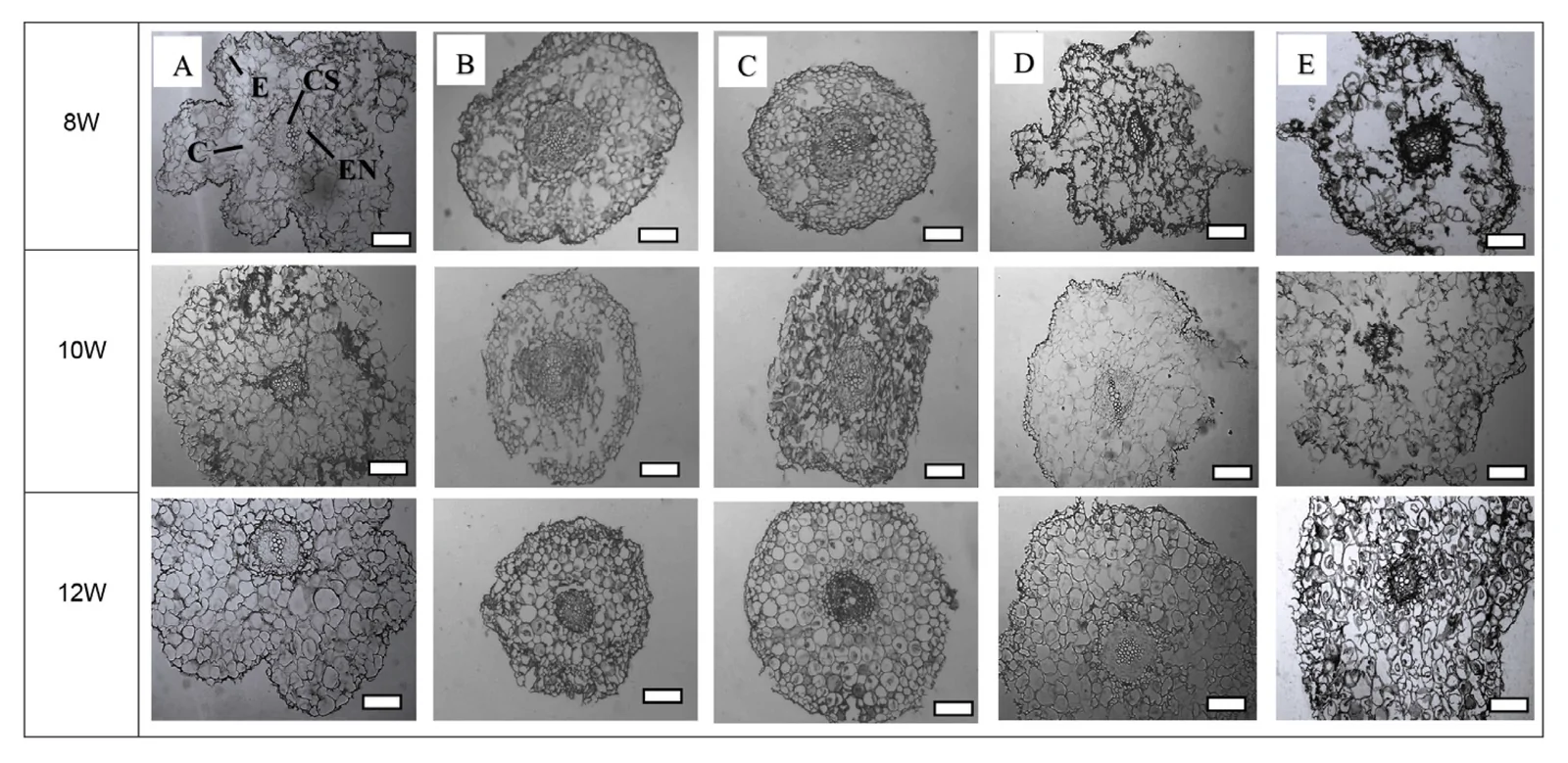
Histological characteristics of *in vitro E. longifolia* hairy root cultures in different LEDs treatments: at 8 weeks, 10 weeks and 12 weeks’ interval. C-cortical, CS-central stele, E-epidermis and EN-endodermis. (Scale bar = 200 μm)

**FIGURE 2:**
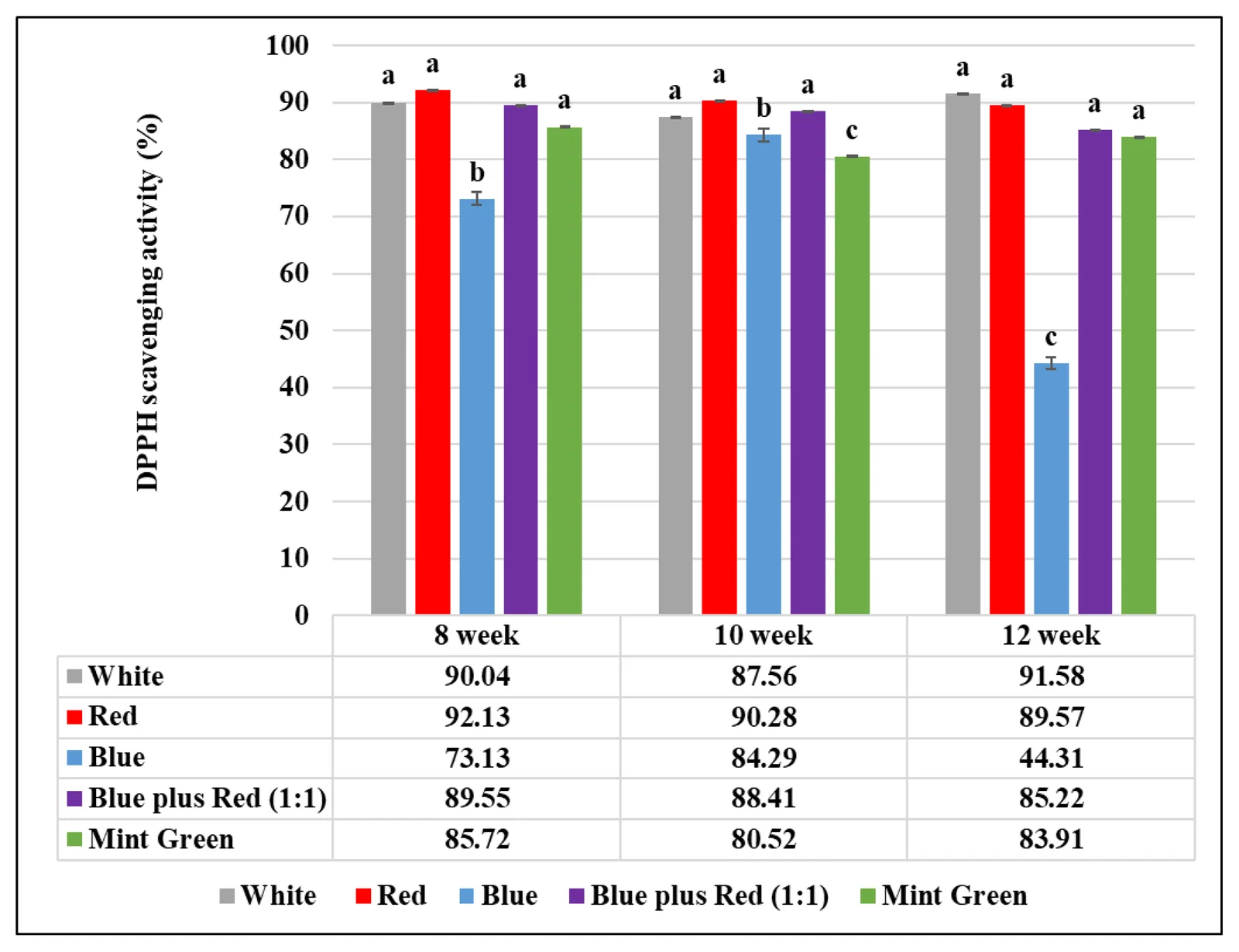
The effect of different LED treatments on radical scavenging activity (DPPH) in *E. longifolia* hairy root cultures at 8 weeks, 10 weeks and 12 weeks. Each week was analysed separately. Bars represent the mean ± standard error (*n* = 3). Means with different letters within the same week indicate significant differences by Duncan’s multiple range test at (*P* ≤ 0.05).

**TABLE 1:** Longitudinal quantitative anatomical measurements (μm) of *E. longifolia* hairy root tissues under different LED treatments.

Week	Light emitting diodes treatment	Cortical width (μm)	Vascular radius (μm)
8	White	288.61 ± 15.68	105.78 ± 3.17
	Red	190.24 ± 4.14	107.57 ± 3.20
	Blue	270.13 ± 8.30	131.51 ± 2.92
	Blue plus Red (1:1)	303.37 ± 7.40	56.56 ± 3.41
	Mint green	250.13 ± 10.93	83.46 ± 1.77
10	White	386.54 ± 15.13	110.22 ± 4.42
	Red	232.14 ± 6.15	112.91 ± 2.61
	Blue	210.01 ± 10.33	122.63 ± 2.24
	Blue plus Red (1:1)	289.43 ± 6.12	80.58 ± 1.17
	Mint green	445.79 ± 13.90	53.27 ± 1.53
12	White	371.79 ± 18.16	100.82 ± 2.70
	Red	313.80 ± 4.25	93.41 ± 2.20
	Blue	234.32 ± 4.05	75.42 ± 1.54
	Blue plus Red (1:1)	395.97 ± 7.06	101.02 ± 0.78
	Mint green	400.24 ± 8.17	64.09 ± 1.00

*Notes*:

* Data represent the mean ± SE of five radial spatial replicates per representative micrograph to account for anatomical variance. Within-micrograph trends are described relative to the White (W) control.

**TABLE 2:** *E. longifolia* hairy root cultures protein and carbohydrate contents response to a different of LEDs.

Week	Light emitting diodes treatment	Protein (mg/mL)	Carbohydrate (mg/mL)
8	White	0.707 ± 0.07 a	70.62 ± 5.48 ab
	Red	0.298 ± 0.01 c	80.86 ± 3.27 a
	Blue	0.271 ± 0.02 c	94.72 ± 0.85 a
	Blue plus Red (1:1)	0.706 ± 0.01 a	31.40 ± 0.30 c
	Mint green	0.567 ± 0.04 b	56.07 ± 2.90 bc
10	White	0.613 ± 0.06 a	65.41 ± 6.62 b
	Red	0.340 ± 0.01 c	83.88 ± 3.28 a
	Blue	0.265 ± 0.03 c	53.40 ± 17.10 b
	Blue plus Red (1:1)	0.616 ± 0.02 a	56.21 ± 9.21 b
	Mint green	0.452 ± 0.06 b	63.38 ± 2.05 b
12	White	0.647 ± 0.06 a	60.61 ± 6.71 b
	Red	0.234 ± 0.01 b	116.79 ± 4.34 a
	Blue	0.275 ± 0.03 b	103.68 ± 15.15 a
	Blue plus Red (1:1)	0.577 ± 0.01 a	78.58 ± 8.21 b
	Mint green	0.652 ± 0.03 a	67.03 ± 1.0 b

*Notes*:

* Values are shown in mean ± standard error. Each week were analysed separately. Within columns, mean ± standard error with the same alphabet letters indicates no significant difference between means by Duncan’s Test, at *p* ≤ 0.05.

## Data Availability

All the data supporting the findings of this study are available within the paper.

## Abbreviations

LEDs: light emitting diodes
ELHRCs: *E. longifolia* hairy root cultures
MS: Murashige and Skoog media
FAA: Formalin
Acetic acid: 95% alcohol
TBA: tert-Butyl alcohol
